# Acceptability and feasibility of oral swabs for tuberculosis diagnosis in young children: a qualitative study from Uganda and Peru

**DOI:** 10.3389/ftubr.2026.1726647

**Published:** 2026-02-12

**Authors:** Francesca W. Basile, Rodrigo Calderón-Flores, Franziska Held, Luisa Pollmeier, Emmanuel Nasinghe, Godfrey Bagenda, Lazaaro Mujumbusi, Rose Namaganda, Angel Kanyange, Prossy Mbekeeka, Abner Tagoola, Jerrold J. Ellner, Susan E. Dorman, Moses Joloba, Carlos Zamudio, Adeodata Kekitiinwa, Rinn Song, Nora Engel

**Affiliations:** 1 Oxford Vaccine Group, Department of Paediatrics, University of Oxford, Oxford, United Kingdom; 2 Instituto de Medicina Tropical Alexander von Humboldt, Universidad Peruana Cayetano Heredia, Lima, Peru; 3 Department of Health Ethics and Society, Faculty of Health Medicine and Life Sciences Maastricht University, Maastricht, Netherlands; 4 Integrated Biorepository of the H3Africa Uganda, Kampala, Uganda; 5 Department of Anatomy, College of Health Sciences, Makerere University, Kampala, Uganda; 6 Department of Immunology and Molecular Biology, College of Health Sciences, Makerere University, Kampala, Uganda; 7 Baylor College of Medicine Children's Foundation–Uganda, Kampala, Uganda; 8 Department of Pediatrics, Jinja Regional Referral Hospital, Jinja, Uganda; 9 Department of Medicine, Rutgers New Jersey Medical School, Newark, NJ, United States; 10 Medical University of South Carolina, Charleston, SC, United States; 11 Faculty of Science, Athena Institute, Vrije Universiteit Amsterdam, Amsterdam, Netherlands

**Keywords:** acceptability, childhood TB, diagnostic research, feasibility, implementation, qualitative study, TB diagnosis delay, tongue swabs

## Abstract

**Background:**

Tuberculosis (TB) remains a leading cause of morbidity and mortality in young children, particularly in settings with limited diagnostic capacity. Oral swabs represent a promising alternative specimen type due to their ease of collection, but evidence regarding their acceptability and feasibility remains limited.

**Methods:**

This qualitative sub-study was nested within the NOD-pedFEND diagnostic trial evaluating novel tests for pediatric TB. We conducted semi-structured interviews and focus group discussions with 81 participants across Uganda (*n* = 57) and Peru (*n* = 24), including caregivers (Uganda, *n* = 30; Peru, *n* = 7), healthcare workers (Uganda, *n* = 23; Peru, *n* = 12), and National TB Program stakeholders (Uganda, *n* = 4; Peru, *n* = 5). Participants were recruited purposively from among those involved in or linked to the parent NOD-pedFEND study. Data were analyzed using thematic analysis.

**Results:**

Oral swabs were widely perceived as acceptable due to their non-invasive nature, minimal discomfort, and ease of collection. Caregivers and healthcare workers valued the reduced burden on children compared to more invasive sampling methods. Across both countries, participants expressed concerns about perceived low diagnostic sensitivity in children, particularly when compared with reference standard specimens. Despite these reservations, oral swabs were welcomed as a complementary, rather than substitute, diagnostic modality. Stakeholders highlighted their potential role within future point-of-care diagnostic strategies in low-resource settings.

**Conclusions:**

Oral swabs are acceptable and feasible for pediatric TB diagnosis in diverse settings, though concerns about sensitivity persist. Their integration as an add-on test could expand diagnostic access, especially if incorporated into scalable, point-of-care approaches.

## Introduction

Tuberculosis (TB) remains a leading cause of morbidity and mortality among children, particularly in resource-limited settings where access to timely and accurate diagnosis is often constrained. In 2024, an estimated 10.7 million people developed TB, of whom were 11% children and young adolescents (aged 0–14 years old), although approximately half of pediatric TB cases remain undiagnosed and untreated (1).

Diagnosing TB in children poses unique challenges due to non-specific symptom presentation, low bacterial loads, and the difficulty in obtaining quality respiratory samples, especially in young children under five (2). As a result, many pediatric cases go undiagnosed or are diagnosed too late. This occurs frequently in decentralized and primary care settings, where diagnostic infrastructure is often limited and pediatric respiratory sample collection techniques such as gastric aspirate (GA) are not feasible (3).

In response, recent pediatric TB diagnostic research has prioritized the development of point-of-care tests and the use of less invasive specimens as critical strategies to improve case detection in children. Stool, for instance, has emerged as a practical alternative to respiratory specimens, enabling microbiological confirmation without the need for invasive procedures such as GA or nasopharyngeal aspirate (NPA) (4).

Oral swabs (OS) similarly represent an easily collectible, non-invasive sample type, particularly suitable for collection in non-sputum-producing populations, such as young children. Current evidence suggests that OS may have lower sensitivity than traditional respiratory specimens, including stool, for the detection of pediatric TB (5, 6). However, their operational simplicity and ease of use may offset this limitation due to a high diagnostic yield, especially if used in combination with other diagnostic tools or as an initial testing method in decentralized multicomponent strategies (7, 8).

As with all novel diagnostic approaches, analytical validation is essential yet not sufficient to ensure successful implementation in real-world settings (9). In pediatric TB, where evidence on the feasibility and acceptability of diagnostic tools in routine care remains limited, this aspect is particularly relevant (10). Frontline TB services often operate under resource constraints, with caregivers and healthcare providers facing competing trade-offs that shape whether and how new tools can be adopted. Understanding the perspectives of these end users is therefore critical, not only to assess acceptability, but also to anticipate practical barriers, align diagnostic procedures with existing workflows, and ultimately support the integration of promising tools into decentralized TB care (11).

Therefore, we aimed to explore the feasibility and acceptability of OS collection and processing for TB diagnosis in children under five in Uganda and Peru—two high-TB-burden countries—focusing on the perspectives and lived experiences of caregivers and healthcare workers (HCWs) to inform the potential implementation of this approach in pediatric TB diagnostic services.

## Methods

### Study design and participants

This qualitative sub-study was nested within NOD-pedFEND, a diagnostic cohort study conducted in Uganda and Peru evaluating novel tests in children under five with presumed TB. The parent study protocol has been described elsewhere (12). In brief, baseline assessments included clinical, radiological, and laboratory evaluations. TB was confirmed using a microbiological reference standard of Xpert MTB/RIF Ultra (Xpert Ultra; Cepheid, Sunnyvale, USA) and/or culture from GA, NPA, or stool. OS were collected over the tongue, cheeks, and sublingual area for 10–15 s. Children abstained from eating, drinking, or toothbrushing for at least 1 h beforehand. OS were obtained prior to GA or NPA, transported to reference laboratories, and tested with Xpert Ultra. Detailed procedures and interim diagnostic results are reported elsewhere (5).

The qualitative sub-study explored the implementability of different TB diagnostic sampling procedures, including OS. Between May 2024 and March 2025, semi-structured interviews and focus group discussions (FGDs) were conducted with (1) caregivers of children enrolled in NOD-pedFEND; (2) HCWs (research nurses, counselors, laboratory staff, clinicians) involved in the parent study; and (3) National TB Program (NTP) stakeholders. Purposive sampling was used to ensure inclusion of participants with direct experience relevant to the study aims. Caregivers were recruited from among children enrolled in NOD-pedFEND who had undergone the study procedures; in Uganda, we also sought to include caregivers of both HIV-positive and HIV-negative children to reflect the clinical diversity of the study population. Healthcare workers were purposively selected based on their involvement in the diagnostic study (including nurses, clinicians, counselors, and laboratory personnel). NTP stakeholders were identified by the study investigators as individuals with responsibility for pediatric TB policy or implementation. Caregiver education level was not collected. Differences in caregivers' participant numbers between Uganda and Peru reflect variation in enrollment rates in the parent study at the time of qualitative data collection rather than purposive balancing across countries. All approached individuals agreed to participate to the study. Data collection and analysis proceeded iteratively, with transcripts reviewed in parallel with ongoing interviews. After every 5–6 interviews within each participant group, the research team assessed whether new data generated additional codes or added new meaning or nuance to existing themes. Saturation was considered reached when consecutive interviews produced no new codes and no further thematic insights, at which point recruitment for that group was concluded (13, 14).

### Study sites

Interviews and FGDs were conducted at a government-owned regional referral hospital located in a peri-rural area and at a large urban public–private HIV/TB clinic in Uganda, as well as at two tertiary-level hospitals in metropolitan Lima, Peru.

### Data collection and analysis

Three interview guides were developed a priori for each category of participants (caregivers, HCWs, NTP stakeholders) and reviewed by the study team and local investigators to ensure question clarity, local context appropriateness, and robustness of the interview guide to address all study objectives. First, we (FH, LP, FWB, NE) generated the guides used in Uganda, which were then revised and translated for use in Peru, ensuring appropriateness to the local context (FWB and RC). The interview guides were informed by the domains of the Health Equity Implementation Framework (15). Interviews were conducted by FWB, RC, FH, and LP and focused on the experiences with the diagnostic journey, multiple sample collection (GA, NPA, stool, urine, saliva, blood, OS), and views on the feasibility, scalability, and implementability of pediatric TB diagnostics. Discussions of diagnostic accuracy and test sensitivity with caregivers were not prompted using technical terminology. Caregivers were not asked directly about “accuracy” or “sensitivity,” and interviewers framed questions around whether testing helped caregivers “know what the child was suffering from,” encouraging reflection on experiences throughout the diagnostic journey, and comparisons between sampling approaches. Therefore, perceptions of test performance emerged spontaneously, typically framed in experiential terms such as whether a test “showed the illness,” “worked,” or “picked the TB,” especially after previous tests had failed.

All participants were asked about OS, and this paper presents the material relevant to the analysis of their acceptability and feasibility. Caregivers who participated in the qualitative interviews had children enrolled in NOD-pedFEND and therefore also contributed quantitative diagnostic accuracy data, as OS collection formed part of the main study protocol. The qualitative research team (FWB, RC, FH, LP, GB, LM) operated independently from the clinical and laboratory teams responsible for sample collection and diagnostic procedures and did not participate in patient care activities.

All interviews were audio recorded. All interviews with participants from Peru were conducted in Spanish. In Uganda, FGDs in Luganda and Lusoga were conducted with the help of translators with prior experience in qualitative research (GB and LM). The remaining interviews took place in English. Due to time and availability constraints, nine interviews were conducted remotely over Zoom, two in English and seven in Spanish.

This oral-swab–focused analysis represents a secondary thematic analysis of the broader qualitative sub-study embedded within NOD-pedFEND. We undertook an inductive thematic analysis in NVivo (v14 for Mac). Initial codes were developed directly from the data, guided by our research question on experiences with and views on OS. Codes and their definitions were formalized into a codebook (Supplementary Annex 1).

The parent qualitative dataset had been coded collaboratively by multiple researchers through iterative independent coding and consensus meetings, resulting in a refined shared codebook. Spanish interviews were transcribed in the original language (FWB, RC). Interviews conducted in Luganda and Lusoga were transcribed and translated into English by bilingual team members (LM, GB) familiar with the study context to ensure accuracy and preservation of meaning, and these English translations were used for coding. Interviews originally conducted in English were coded as transcribed (FH, LP). Coding therefore took place in Spanish or English depending on the interview language.

For the present analysis, FWB coded all oral-swab–related transcripts using this refined codebook as a foundation. Additional inductive codes were developed when OS-specific insights emerged, and these were reviewed in analytic discussions with other qualitative team members (RC, NE, FH, LP) and incorporated into the overarching codebook. Through iterative review and discussion, these codes were organized into higher-level themes that represented patterns across the dataset.

Credibility of the findings was enhanced through data source triangulation, drawing on interviews and FGDs with caregivers, healthcare workers, and NTP stakeholders across both Uganda and Peru. Regular consensus meetings among the multidisciplinary research team supported reflexive discussion of coding decisions and interpretation of emerging themes, functioning as an internal form of peer debriefing. Member checking was not undertaken due to logistic constraints within the clinical study context.

The research is reported in accordance with the COnsolidated Criteria for REporting Qualitative research checklist (COREQ, Supplementary Annex 2) (16).

### Ethics

The study was approved by the Institutional Review Boards of the University of Oxford, Makerere University, Universidad Peruana Cayetano Heredia, and Rutgers University. All participants took part in the study after providing informed consent. Participant information forms translated in the local languages (Luganda, Lusoga) and Spanish were provided or read to participants to enable them making informed decisions. Literate participants provided written consent, and those who were unable to write used a thumb print and an impartial witness signed.

## Results

### Participant overview

A total of 81 participants from Uganda and Peru were recruited for individual interviews and FGD (Table 1). These comprised 37 caregivers (30 in Uganda and 7 in Peru), 37 HCWs (23 in Uganda and 14 in Peru), and 7 NTP stakeholders (4 in Uganda and 3 in Peru).

**Table 1:** Participant characteristics by country.

**Participants**	**Uganda**	**Peru**
Healthcare workers, *n* (female)	23 (11)	12 (11)
NTP stakeholders, *n* (female)	4 (2)	5 (3)
Caregivers, *n* (female)	30 (29)	7 (6)
Median age (range)	33 (22–68)	36 (22–61)
Total participants	**57**	**24**

**Figure 1:**
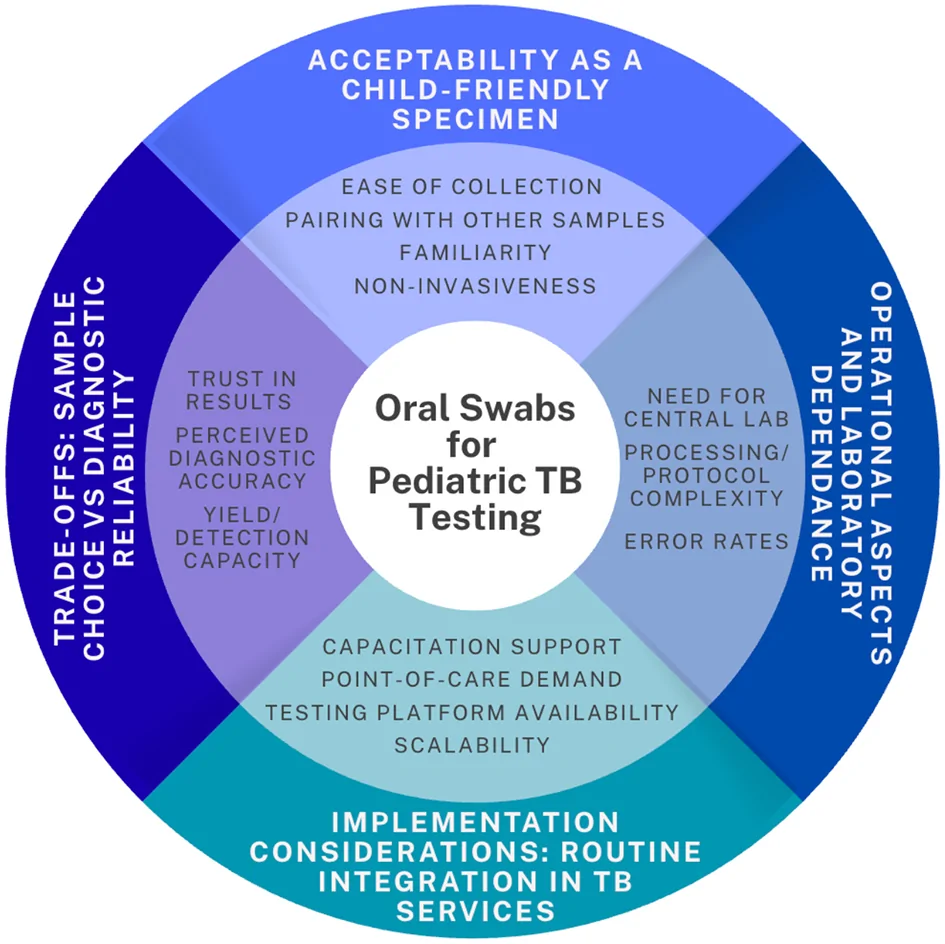
Thematic wheel map of findings from interviews and focus group discussions on oral swabs for pediatric TB diagnosis. The four quadrants illustrate the main themes identified: acceptability of swabs as a child-friendly specimen, trade-offs in specimen choice vs. diagnostic reliability, operational aspects of laboratory dependence, and implementation considerations for routine integration in TB services. Within each quadrant, key subthemes raised by participants are summarized, ranging from collection ease and familiarity to concerns about diagnostic accuracy, laboratory requirements, and the scalability of oral swab testing.

The analysis generated four overarching themes, each encompassing several subthemes that reflected different aspects of participant perspectives on OS. Figure 1 illustrates the relationships between these themes and subthemes.

### Acceptability of OS as a child-friendly diagnostic specimen

Caregivers and HCWs described OS as a non-invasive, painless, and quick sample. These features contributed to a generally high acceptability of this specimen. Some caregivers expressed relief that the swab procedure did not distress their children, particularly when compared to more invasive methods such as GA or NPA.

*I think in this case, swabs do work—because they're something that doesn't really scare the patient. In fact, they often find it funny; they're not that afraid because we explain it to them: “Look, it's something flexible, it moves, it's not going to hurt you, it won't cause pain,” right? — Nurse, Peru*

Caregivers and nurses perceived that older children were very cooperative during OS collection, and while some mild discomfort was noted in infants, this remained manageable. Some study nurses reported difficulties in OS collection among specific populations, such as severely malnourished children, but these were considered minor compared with the procedural challenges associated with sputum, GA, or NPA collection.

*So, for the younger ones, let's say less than two years, they are likely to feel very uncomfortable, like having things in their nose, nostrils, things in their mouth, something that tastes weird, maybe that is… well, they will expect maybe something tasty in the mouth, but then it is something that feels weird, feels prickly, maybe. — Clinician, Uganda*

Familiarity with swabs from prior diagnostic experiences emerged as an important factor shaping caregivers' understanding and acceptance of the procedure. Some HCWs and caregivers in both countries referenced their COVID-19 pandemic experiences with OS.

*The oral swabs are also good because people have that prior knowledge of COVID. So, you tell them that one that was done during COVID, they can say, “Ok.” — Clinician, Uganda*

### Trade-offs in specimen choice and diagnostic reliability

While OS were preferred over invasive procedures due to comfort and ease, both HCWs and caregivers expressed skepticism about their diagnostic sensitivity, especially for young or severely ill children. Some caregivers viewed the method as too superficial and were concerned it might miss the bacilli located deeper in the lungs.

*I prefer the swab. Of course. It's simpler. But if it's about going deeper, it's the one up here [points to nose], because it goes inside and gives more reliable results. But in the moment, I suffer, because it's my child—so I prefer the mildest option. But if there's better information and a treatment to rule it out, then it's the one up here that I prefer [points to chest], because it goes inside. That's how it is. — Caregiver, Peru*

Caregivers and HCWs were willing to perform multiple procedures to ensure their child's illness was accurately diagnosed. Some stakeholders highlighted the need for parallel testing strategies combining OS with other sample types to improve confirmation rates.

*But well, I'm convinced that we need to look for as many diverse methodologies as possible to make bacteriological confirmation easier in young children. And oral swabs, without a doubt, are something we should start with. — NTP program officer, Peru*

While some HCWs acknowledged that OS testing might have lower diagnostic sensitivity compared to other methodologies, participants in both countries emphasized the value of having any test that is child-friendly and operationally feasible, especially considering the limited diagnostic options for young children.

Some NTP stakeholders regarded OS as an imperfect but better-than-nothing tool—an option they would consider, especially when other methods are not viable, such as in primary healthcare facilities where the collection of invasive samples is not feasible.

*Right now, the way things are, I'd say… we don't have many alternatives for… for diagnosing a group of children… we're basically left with nothing. It's like you're giving me a test with zero sensitivity. I mean, I'd rather have a test with 20% sensitivity than one with zero—even more so if it's noninvasive. So, if you ask me what I think, yes. — NTP advisor, Peru*

### Operational aspects of laboratory-dependent testing

HCWs noted that while OS could be collected easily at the clinic, processing and analysis would still require laboratory infrastructure and trained personnel, raising concerns about where testing would take place.

*I think with the oral swabs, okay, they're doable, but then the analysis of these swabs. Who's doing this? Because you, you can take the swabs [at the clinic], but then, you send it to the lab, right? — Nurse, Uganda*

In both countries some laboratory technicians expressed concerns about contamination risks and error rates. The main concerns focused on the buffer preparation and heat inactivation steps required by the study-specific protocol, which were considered challenging for scale-up in peripheral or lower-tier laboratories. Laboratory staff also noted occasional technical difficulties during sample processing—for example, when swabs were mixed (“vortexed”) in buffer, debris sometimes clogged the Xpert cartridge.

*When vortexing swabs, particles sometimes blocked the cartridge. — Laboratory technician, Peru*

### Implementation considerations for the integration of OS into routine services

Despite the sample processing limitations mentioned above, in both countries there was a widespread interest among NTP stakeholders and HCWs in supporting the integration of OS in pediatric TB services.

HCWs in both countries found that the OS had key characteristics for integration into routine outpatient care: it was quick to administer, well accepted by the parents and tolerated by the children, and did not require extensive preparations or expertise.

*The potential [of OS] is big and if it can work, that will be a game changer, big one. You still need something that, so that I don't have to go to the testing center somewhere. If I can have it on my ward in the bedside or in the side room, pick the swab like you do a malaria test. — NTP stakeholder, Uganda*

However, the limited availability of Gene Xpert platforms for use on non-sputum samples was mentioned as a potential barrier for large-scale implementation of OS testing in Peru.

*Another barrier is access to the tests—there are few, but they are available. Like I was saying a moment ago, we have a significant number of Xpert machines, but they're not used for other types of samples besides sputum or gastric aspirate. — NTP advisor, Peru*

Additionally, participants expressed the desire for portable, real point-of-care instruments for OS testing.

*[If] you can innovate a smaller machine, then this is going to be the real true point-of-care test. It will be a game changer. — NTP advisor, Uganda*

In both countries, HCWs noted that prior experience with COVID-19 testing facilitated rapid uptake of the OS procedure. In Uganda, participants also underscored the need for facilitation and support from the Ministry of Health, particularly for capacity building and provision of equipment.

*What would be needed is that the Ministry capacitates the staff and provides equipment to process the sample. — Program officer, Uganda*

## Discussion

This study explored the feasibility and acceptability of OS testing for TB diagnosis in children under five, based on qualitative data from 81 caregivers, HCWs, and NTP stakeholders in Uganda and Peru.

Our findings indicate that in both countries OS were widely regarded as a highly acceptable and feasible diagnostic sample, particularly due to their non-invasive nature, procedural simplicity, and compatibility with primary healthcare and community settings.

Caregivers valued OS for their minimal discomfort, while HCWs emphasized ease of collection and limited training needs, often referencing familiarity with COVID-19 swabbing. Importantly, participants mentioned that such benefits would extend beyond referral hospitals, suggesting potential scalability to lower-tier facilities and active case finding campaigns. These views are especially relevant in pediatric populations where traditional diagnostic approaches, such as GA or NPA, are technically demanding, distressing to children and caregivers, and logistically challenging (10). In contrast to other minimally invasive samples such as stool and urine, which can introduce delays when specimens cannot be collected during the visit and may carry some stigma (17, 18), OS were viewed as immediately obtainable, eliminating the need for return visits and supporting more streamlined, same-encounter diagnostic pathways. Additionally, the encouraging findings on OS acceptability in young children suggest potential for evaluating caregiver-collected swabs in this age group, as well as self-swabbing approaches among older children and adolescents (19, 20).

Despite the positive experiences with sample collection, some HCWs raised concerns about the low diagnostic sensitivity of OS. These concerns appeared to draw on general impressions or prior professional knowledge, as HCWs were blind to the diagnostic accuracy results within this study. Caregivers echoed similar doubts, though for different reasons, often perceiving OS as “too superficial” to replace reference standard specimens. Such views align with current literature, which reports a sensitivity of OS testing in children ranging from 6% to 30%, depending on age group and testing modality (5, 6). Importantly, participants in our study were not informed of the OS diagnostic accuracy results from the parent study. Their skepticism therefore reflects perception-based judgments—often grounded in intuitive beliefs about where TB bacilli “sit” in the body and which procedures feel sufficiently deep to detect them. Evidence from pediatric respiratory diagnostics shows similar patterns: in one COVID-19 study, caregivers preferred saliva sampling for comfort but were willing to accept a more invasive nasopharyngeal swab if they believed it to be more accurate, illustrating that perceived diagnostic performance can outweigh discomfort (21). This highlights how end-user expectations and real-world interpretations of test performance may influence acceptance and uptake independently of formal diagnostic accuracy estimates.

Nonetheless, OS were generally welcomed as an add-on to more invasive samples, and NTP stakeholders emphasized the importance of tools that—while not perfect—fit the realities of care delivery in low-resource settings. Such views reflect an implementation-oriented mindset among frontline workers and decision-makers and are particularly meaningful as the concept of diagnostic yield gains momentum in the scientific community (7). Modeling studies suggest that imperfect but scalable tests can still enhance case detection when used strategically (22). Moving forward, skepticism among caregivers and HCWs underscores the need for further pediatric research to improve the diagnostic accuracy of OS testing, evaluate the performance of minimally invasive combinations such as OS–stool, and develop yield models grounded in pediatric-specific data.

Although caregiver and HCW experiences with OS were broadly similar across Uganda and Peru, also reflecting shared familiarity with swab-based testing introduced during the COVID-19 pandemic and the structured counseling provided within the diagnostic trial, several contextual differences shaped how participants viewed future implementation pathways.

A key insight was the perceived potential of OS testing for point-of-care application. However, several potential barriers to implementing OS-based diagnostics at scale were noted. While the study procedures took place in well-resourced research laboratories in both countries, Peruvian laboratory staff highlighted the gap between research capacity and routine programmatic infrastructure, particularly in peripheral facilities where training, biosafety systems, and equipment remain limited. Ugandan participants similarly recognized infrastructural constraints, but their concerns centered more on access to portable or point-of-care devices that could reduce reliance on centralized laboratories, especially in remote districts. These findings emphasize that laboratory fine-tuning remains a key aspect of future research on OS, as variability in swab processing and the occurrence of non-actionable results reflect valid concerns raised by HCWs and highlight the need for optimized, standardized protocols (23). Future development of streamlined workflows, including single-step lysis buffers, swab-to-cartridge formats, or protocols requiring minimal manipulation, could support scale-up in peripheral or lower-tier laboratories where staffing and biosafety capacity are limited.

Stakeholders highlighted the transformative potential of bedside use, describing OS as a “game changer” if paired with accessible diagnostic platforms. At the same time, the existence of important barriers was raised. In both settings, participants viewed OS as compatible with routine outpatient workflows, yet emphasized different system-level steps needed to enable implementation—policy authorization in Peru and decentralization support in Uganda.

In Peru, the restricted use of GeneXpert machines for non-sputum samples was seen as a major constraint, and across both countries there was a call for portable, true point-of-care instruments tailored for OS testing. These perspectives point to the wish for simplified, decentralized testing workflows (that may include portable, battery-operated diagnostic devices if found feasible) to realize the potential of diagnostic innovation for care delivery (24). Recent multicountry evidence demonstrates that swab-based molecular testing can be successfully paired with near-point-of-care platforms that are portable, battery-operated, and designed for use in peripheral facilities, while meeting WHO accuracy targets for non-sputum point-of-care testing (25). Although these technologies were not evaluated in this study, participants' emphasis on near-patient testing underscores the relevance of pairing an acceptable specimen type with more flexible diagnostic platforms. Ugandan stakeholders, in contrast, focused more on the feasibility of decentralizing testing and on the need for Ministry-led capacity building to support the integration of novel diagnostic procedures into lower-tier facilities. As with other novel TB diagnostics, in both countries training and institutional support were emphasized, and Ministry of Health involvement was viewed as essential for building capacity and ensuring sustainable and context-appropriate implementation strategies (26).

Together, these insights highlight concrete opportunities for aligning technical innovation with health-system readiness to maximize the impact of minimally invasive pediatric TB diagnostics.

Future implementation research will benefit from applying established implementation frameworks to more systematically explore the organizational, contextual, and health-system factors that stakeholders identified as critical for the scalable integration of OS testing.

This study has both strengths and limitations. A key strength is the inclusion of diverse stakeholders from two high-TB-burden countries. Caregiver and HCW accounts often drew on experiences prior to enrollment in the diagnostic study, providing valuable insight into the real-life diagnostic experiences for children with presumed TB in both countries. We also interviewed NTP officers engaged in service delivery at decentralized and primary care levels. This range of perspectives capturing experiences across both specialized and programmatic environments enhanced the representativeness of the findings and their applicability to different levels of the health system. Nevertheless, the study was nested within a diagnostic accuracy trial, and perspectives were limited to caregivers and providers of children under five. Participants were aware they were taking part in research, which may have shaped their perceptions of the sampling procedures and interactions with staff. This awareness may introduce selection and social desirability bias, and findings may not fully reflect experiences under routine care conditions. Furthermore, the interviews explored multiple diagnostic testing approaches, with OS representing only one of the testing options discussed. Future studies should integrate economic evaluations and real-world implementation pilots to complement these qualitative insights.

## Conclusions

OS were regarded as a promising, acceptable, and feasible tool for pediatric TB diagnosis, particularly well-suited to decentralized and low-resource settings. While concerns about diagnostic sensitivity remain, their high acceptability among caregivers and health care workers highlights their potential value, especially when combined with other minimally invasive sample types such as stool. To realize the full potential of OS for pediatric TB diagnosis, further work is needed to strengthen diagnostic performance, simplify and harmonize testing protocols, and advance user-centered implementation strategies that can be readily adopted in primary healthcare settings.

## Data Availability

The raw data supporting the conclusions of this article will be made available by the authors, without undue reservation.
