# Supplementary material for: Acceptability and feasibility of oral swabs for tuberculosis diagnosis in young children: a qualitative study from Uganda and Peru
Source: Front Tuberc. 2026 Feb 12;4:1726647. doi: 10.3389/ftubr.2026.1726647 (PMC12927657; doi:10.3389/ftubr.2026.1726647)
Supplement: Supplementary file 1 [file Table_1.docx]

**ANNEX 1- Codebook**

| *Parent Code* | *Child Code* | *Definition* | *Example* |
| --- | --- | --- | --- |
| OS Collection | Collection of OS | Includes descriptive, direct experiences of caregivers, HCW involved in NOD-pedFEND study who experienced OS collection. | *“Los hisopados eran los más sencillos, en realidad. Los bebés se abrían la boquita… No había problema.”-* Nurse, Peru  (Swabs were the simplest; the babies opened their mouths, there were no issues)  *“Even me it did not affect me. They would put it and remove it in that very minute. There was no crying or fighting.” –* Caregiver, Uganda |
|  | Collection of OS with other samples | Includes both descriptive, direct experiences of caregivers and HCW with, and views on, parallel/multiple samples collection that includes OS. | *“Me refiero a que se le podría recolectar en un mismo día o quizás… demoraría un día adicional, pero podrían ser muestras que se podrían recolectar más fácil.”- Nurse, Peru*  (It could be collected in one day, or sometimes it might take an extra day, but the samples could still be collected more easily.)  “*Saliva [oral sampling] has no problem but my child had less saliva, so they collected it many times to get what they wanted*.”- Caregiver, Uganda |
|  | Familiarity | Includes prior experiences or knowledge of OS testing (as could be for COVID-19 or other diseases). | *“Ya tenemos experiencia con COVID, entonces era súper sencillo hacer el procedimiento*.”- Nurse, Peru  (We already had COVID experience, so the procedure was easy)  *“Pre-existing knowledge from COVID-19 testing helps in expectation management with caregivers.”-* Counselor, Uganda |
|  | Preference | Includes expressions of choice, liking, or prioritization of OS over other sample types or testing modalities. | *“Prefiero el hisopado. No es tanta contaminación como las heces*.”- Lab technician, Peru  *(I prefer the swab; it’s less contaminated than stool)*  *“Like this one [oral swab] is not painful. But this one in the nose [GA tube] is a little bit painful.”-* Caregiver, Uganda |
|  |  |  |  |
| Laboratory Experiences | Sample processing | Includes hands-on experiences of sample processing in the context of NOD-pedFEND study, strengths and challenges of the operating procedure. | *“Al ser hisopos, quizá al vortexarlo, había partículas que obstruían un poco el cartucho.”-* Lab technician, Peru  (When vortexing swabs, particles sometimes blocked the cartridge.)  *“Oral swabs alone would not be trusted for diagnosing TB because of poor yield; risk of contamination is there (and happened).”-* Lab technician, Uganda |
|  | Xpert testing | Includes views specifically linked to OS testing on the Xpert platform, including availability and implementability of this testing modality. | *“En otros laboratorios creo que mucho Xpert no se trabaja por lo mismo que el costo… me pareció una buena forma de diagnóstico.”-* Lab technician, Peru  (In other labs, Xpert isn’t used much because of the cost… but I thought it was a good way to diagnose.) |
| Trustworthiness and Diagnostic Accuracy | Trust in results | Includes comments about whether participants believe OS test results are reliable, valid, or can be acted upon with confidence. | “Basta que me salga positivo en esa prueba, yo inicio el tratamiento.”- Clinician, Peru (If the test [OS] comes back positive, I start treatment.)  *“When you cough, that thing that comes from the mouth… that is the best thing to test TB.”-* Caregiver, Uganda |
|  | Perceived diagnostic accuracy of TB samples | Includes views related to the comparative sensitivity and/or specificity of OS, compared with other samples. | *“Yo creo que para obtener un resultado bien, el esperado, sería los aspirados y un hisopado.”-* Caregiver, Peru  (To get a proper result, aspirates and swabs would be best.)  *“They can use the nasal swab rather than the NPA. Because if they can detect TB from the nasal swab, then NPA is not needed.”- Clinician, Uganda* |
|  | Yield/detection capacity | Includes comments about the ability of OS testing to be applied to detect enough cases, its usefulness in practice. | “*Si son varios, mucho mejor, porque ya con eso nos están definiendo que sí tiene la enfermedad.”-* Clinician, Peru (If there are several samples, even better, because that confirms the disease.)  *“Oral swabs alone would not be trusted… poor yield.”-* Lab technician, Uganda |
| Implementation | Trainings/  Capacitation | Includes quotes with comments on training needs, both in the context of the study and externally in routine settings/NTP. | *“Quizás hacer tu primera muestra no supiste hacerlo bien, pero conforme vas trayendo más muestras, vas encontrando una técnica…”-* Lab technician, Peru (Maybe the first wasn’t done well, but with more practice you learn the right technique)  *“What would be needed is that the Ministry capacitate the staff and provide equipment to process the sample.”-* Program officer, Uganda |
|  | Point-of-care use | Includes attributes of OS collection and testing that are relevant considerations for point-of-care application. | *“Si fuese así como el GeneXpert… eso sí se podría implementar en las postas, donde necesitamos un rápido diagnóstico*.”- NTP stakeholder, Peru  (If it worked like GeneXpert, it could be used at primary health centers for rapid diagnosis.)  *“What I mean, if there can be a way, you can innovate a smaller machine, then this is going to be the real true point of care test. It will be a game changer.”-* NTP advisor, Uganda |
|  | Scalability | Includes comments with stakeholder views on requirements and barriers for large-scale implementation. | *“Serían más que todo los materiales, porque no llegan todos los materiales… If we don’t have the resources, we cannot do it.”-* NTP stakeholder, Peru  (The main challenge is that the materials don’t always arrive. If we don’t have the resources, we cannot do it)  *“For them [rural centers], trainings. Sometimes they don’t have the necessary implements. They don’t have a permanent laboratorian. They don’t have a GeneXpert.”-* NTP stakeholder, Uganda |

**ANNEX 2- COREQ Checklist**
